# LOXL2 induces aberrant acinar morphogenesis via ErbB2 signaling

**DOI:** 10.1186/bcr3461

**Published:** 2013-08-23

**Authors:** Joan Chang, Monica M Nicolau, Thomas R Cox, Daniel Wetterskog, John WM Martens, Holly E Barker, Janine T Erler

**Affiliations:** 1 Hypoxia and Metastasis Team, Division of Cancer Biology, The Institute of Cancer Research, 237 Fulham Road, London, UK SW3 6JB; 2 Biotech Research & Innovation Centre (BRIC), University of Copenhagen, Ole Maaløes Vej 5, Copenhagen 2200, Denmark; 3 Department of Mathematics, Stanford University, 450 Serra Mall Stanford, CA 94305, USA; 4 Gene Function Team, The Breakthrough Breast Cancer Research Centre, The Institute of Cancer Research, 237 Fulham Road, London, UK SW3 6JB; 5 Department of Medical Oncology, Erasmus MC Cancer Institute and Cancer Genomics Netherlands, Dr Molewaterplein 50, 3015 GE Rotterdam, The Netherlands

## Abstract

**Introduction:**

Lysyl oxidase-like 2 (LOXL2) is a matrix-remodeling enzyme that has been shown to play a key role in invasion and metastasis of breast carcinoma cells. However, very little is known about its role in normal tissue homeostasis. Here, we investigated the effects of LOXL2 expression in normal mammary epithelial cells to gain insight into how LOXL2 mediates cancer progression.

**Methods:**

LOXL2 was expressed in MCF10A normal human mammary epithelial cells. The 3D acinar morphogenesis of these cells was assessed, as well as the ability of the cells to form branching structures on extracellular matrix (ECM)-coated surfaces. Transwell-invasion assays were used to assess the invasive properties of the cells. Clinically relevant inhibitors of ErbB2, lapatinib and Herceptin (traztuzumab), were used to investigate the role of ErbB2 signaling in this model. A retrospective study on a previously published breast cancer patient dataset was carried out by using Disease Specific Genomic Analysis (DSGA) to investigate the correlation of *LOXL2 *mRNA expression level with metastasis and survival of ErbB2-positive breast cancer patients.

**Results:**

Fluorescence staining of the acini revealed increased proliferation, decreased apoptosis, and disrupted polarity, leading to abnormal lumen formation in response to LOXL2 expression in MCF10A cells. When plated onto ECM, the LOXL2-expressing cells formed branching structures and displayed increased invasion. We noted that LOXL2 induced ErbB2 activation through reactive oxygen species (ROS) production, and ErbB2 inhibition by using Herceptin or lapatinib abrogated the effects of LOXL2 on MCF10A cells. Finally, we found LOXL2 expression to be correlated with decreased overall survival and metastasis-free survival in breast cancer patients with ErbB2-positive tumors.

**Conclusions:**

These findings suggest that LOXL2 expression in normal epithelial cells can induce abnormal changes that resemble oncogenic transformation and cancer progression, and that these effects are driven by LOXL2-mediated activation of ErbB2. LOXL2 may also be a beneficial marker for breast cancer patients that could benefit most from anti-ErbB2 therapy.

## Introduction

Lysyl oxidase-like 2 (LOXL2) is one of five members of the lysyl oxidase (LOX) family of extracellular matrix proteins and mediates the cross-linking of stromal collagens and elastin [1-3]. We previously showed that LOXL2 expression is clinically correlated with increased metastasis and poor survival in breast cancer patients with estrogen receptor (ER)-negative tumors [4]. Consistent with this, other studies found LOXL2 protein levels to be higher in poorly differentiated breast carcinomas, and elevated *LOXL2 *mRNA was observed in invasive and metastatic breast cancer cell lines [5,6]. We showed that LOXL2 plays a critical role in breast cancer progression, and further demonstrated that genetic, chemical, or antibody inhibition of LOXL2 significantly reduced the size and number of metastases in the lungs, liver, and bone through blocking the effects of extracellular LOXL2 on matrix remodeling and cell invasion [4]. In accordance with our findings, Barry-Hamilton *et al*. [7] demonstrated efficacy of an LOXL2-targeting antibody in reduction of bone and soft tissue metastases after intracardiac injection of human breast cancer cells [7]. This antibody is now in phase II clinical trials. Upregulation of LOXL2 is also associated with poor prognosis in patients with squamous cell, colon, and esophageal cancers [8,9]. Furthermore, LOXL2 is linked to drug resistance in pancreatic cancer cells [10] and promotes gastric cancer metastasis [11]. Thus, LOXL2 is likely to be an excellent drug target in many cancer types [12].

Despite the mounting evidence suggesting a critical role for LOXL2 in metastasis, very little is known about its function during development or its role in normal tissue homeostasis. *LOXL2 *mRNA was detected at low levels in heart, lung, and kidney, and at high levels in the prostate, uterus, and placenta [13]. However, LOXL2 protein levels were very low in all normal adult tissues [7]. As a result, no deleterious side effects have been observed in response to LOXL2 inhibition [4,7].

In the adult mammary gland, epithelial cells are organized into ducts and lobules. The lobules comprise multiple mammary acini, and each lobule has a mammary duct connecting the lobules to the nipple. Normal acini have lumens formed by a single layer of polarized luminal epithelial cells, surrounded by myoepithelial cells, and finally, the basement membrane. Epithelial cells grown on plastic do not accurately reflect the *in vivo *microenvironment of the mammary gland, highlighting the importance of studying tumorigenesis by using a three-dimensional (3D) model mimicking normal mammary epithelial development.

Studying LOXL2 function in normal cells can provide information about its role in cancer; thus, here we used the well-established MCF10A acini assay [14] as a 3D model to investigate the role of LOXL2 in mammary epithelial acini formation and to provide novel insights into the molecular events regulated by LOXL2.

We showed that overexpression of LOXL2 induces invasive branching structures on cells plated onto extracellular matrix (ECM) and increases invasiveness of the cells. LOXL2 induction also causes aberrant acini development by interfering with apoptosis, proliferation, and polarization, and these LOXL2-mediated effects are attenuated when ErbB2 signaling is inhibited. In this article, we are the first to demonstrate that LOXL2 induces phosphorylation of ErbB2 through the production of reactive oxygen species (ROS). Moreover, we showed that LOXL2 expression correlates with metastasis and poor survival in ErbB2-positive breast cancer patients.

## Materials and methods

### Cell lines and cell culture

All cell lines used in this study were obtained from American Type Culture Collection. Human MCF10A normal mammary epithelial cells were grown in F12/DMEM (Invitrogen) media supplemented with 5% horse serum (HS, Invitrogen), 0.5 μg/ml hydrocortisone (Sigma), 10 μg/ml insulin (Sigma), 100 ng/ml cholera toxin (Sigma), and 20 ng/ml EGF (Peprotech). MCF10A cell lines used were cultured for no more than 20 passages. Human MDA-MB-361 breast cancer cells were grown in F12/DMEM (Invitrogen) media supplemented with 20% fetal calf serum (Invitrogen). Human MDA-MB-231 breast cancer cells were grown in DMEM (Invitrogen) media supplemented with 10% fetal calf serum (Invitrogen).

### Generation of cell lines

For overexpression of the LOXL2 protein, the human *LOXL2 *gene was amplified by polymerase chain reaction (PCR) and cloned into the pBABE retroviral vector by using the following primers: 5'-CGC GGA TCC ATG GAG AGG CCT CTG TGC TCC CAC-3', 3'-CGC GTC GAC TTA CTG CGG GGA CAG CTG GTT GTT TAA GAG C-5'. MCF10A cell line was infected with retroviruses expressing the pBABE vector containing *LOXL2 *(10A L2), or parental pBABE retroviral vector as a control (10A cont).

### Quantitative real-time PCR

Total RNA was isolated from cell pellets by using RNeasy Mini Kits (Qiagen). RNase treatment and cDNA synthesis were done by using RNaseOUT and M-MLV Superscript (Invitrogen), according to manufacturer's instructions. *β-actin *was used as an internal control. The following primers were used for the analysis of *LOXL2*, β-actin, E-cadherin, Vimentin, SNAI1, SNAI2, α-SMA, TWIST, and N-cadherin: LOXL2 forward, 5'-CTG CCA CAT AGG TGG TTC T-3'; LOXL2 reverse, 5'-TGG CAT TCG TTC AGA CTC AG-3'. β-actin forward, 5'-GAG GCC CAG AGC AAG AGA GG-3'. β-actin reverse, 5'-TAC ATG GCT GGG GTG TTG AA-3'. E-cadherin forward, 5'-TGG AGG AAT TCT TGC TTT GC-3'; E-cadherin reverse, 5'-CGC TCT CCT CCG AAG AAA C-3'. Vimentin forward, 5'-CTT CGC CAA CTA CAT CGA CA-3'; Vimentin reverse, 5'-CGG CCA GCA GGA TCT TAT T-3'. SNAI1 forward, 5'-AGG ATC TCC AGG CTC GAA AG-3'; SNAI1 reverse, 5'-TCG GAT GTG CAT CTT GAG G-3'. SNAI2 forward, 5'-TCC GAA GCC AAA TGA CAA AT-3'; SNAI2 reverse, 5'-TGT GTG TGC ATA TGT GTG TGT G-3'. α-SMA forward, -5'-CCC TGA AGT ACC CGA TAG AAC A-3'; α-SMA reverse, -5'-GGC AAC ACG AAG CTC ATT G-3'. TWIST forward, 5'-GGG CCG GAG ACC TAG ATT-3'; TWIST reverse, 5'-TTC TGA ATT GTA TCA CAC CTT CTC A-3'. N-cadherin forward, 5'-GTG CTC AGG CTG TGG ACA TA-3'; N-cadherin reverse, 5'-CTG CAC TTT GAT GAT GAA TTC TG-3'.

The following reaction cycle was used: 95°C for 10 minutes, followed by 40 cycles of 95°C for 15 seconds, 60°C for 30 seconds, 72°C for 30 seconds, and a final step of 72°C for 10 minutes. The PCR was performed by using an Applied Biosystems 7900HT Fast Real-Time PCR system (Applied Biosystems, Carlsbad, CA, USA), or Roche LightCycler480II (Roche Applied Science).

### 2D and 3D proliferation assays

The 2D proliferation on plastic and 3D proliferation in Matrigel of the cells were assessed by using the CellTiter 96^® ^AQ_ueous _One Solution Cell Proliferation Assay (Promega), according to the manufacturer's instructions. Cells were plated in triplicate in 96-well plates at 2,000 cells per well, either in 100 μl of media or in 50 μl of Matrigel (BD Biosciences). All readings were normalized to day 0 readings.

### Western blotting

All cells were lysed in 1% NP40 buffer supplemented with 10 μg/ml aprotinin (Sigma) and 10 μg/ml leupeptin (Sigma).

Conditioned medium (CM) was obtained as previously described [15]. For lysate preparation, 350,000 cells were plated on Matrigel-coated six-well plates. After overnight incubation at 37°C, 5% CO_2_/20% O_2_, the cells were serum-starved for 3 hours and then serum-blasted for 15 minutes before collection for maximal signaling activation.

For drug treatments, cells were plated out as described earlier, and 3 hours after plating, the cells were exposed to lapatinib or Herceptin at the indicated concentrations. Equivalent amounts of DMSO or human kappa IgG (Sigma) were added to the cells as a control for lapatinib and Herceptin, respectively. After overnight incubation with the drugs, the cells were serum-starved for 3 hours and then serum-blasted for 15 minutes in presence of drug before collection.

For catalase treatment with or without recombinant human LOXL2 (rhLOXL2), the cells were plated as described earlier. After overnight incubation in the absence of treatment, the cells were serum-starved for 3 hours and then subjected the following combination of treatments for 15 minutes before collection: 200 U/ml catalase (Sigma), 50 n*M *recombinant human LOXL2 (rhLOXL2, R&D Systems), 200 U/ml catalase, and 50 n*M *rhLOXL2.

For rhLOXL2 treatment only, 700,000 cells were plated on polyacrylamide-crosslinked 50-mm-diameter coverslips that were coated with Matrigel by using a previously published protocol [16]. The 50 n*M *rhLOXL2 was added onto one set of 10A control cells. After overnight incubation at 37°C, 5% CO_2_/20% O_2_, the cells were serum-starved for 3 hours and then serum-blasted for 15 minutes before collection.

Proteins were separated with NuPAGE Novex Bis-Tris 10% gels or 7% Tris-Acetate gels (Invitrogen), transferred to polyvinylidene difluoride membranes (Millipore), and immunoblotted with antibodies specific for human ErbB2, pErbB2, EGFR, pEGFR, Erk1/2 (MAPK), pErk1/2 (pMAPK), Akt, and pAkt (all Cell Signaling), or human LOXL2 (Abcam). Hsc70 or β-actin (Abcam) was used as a loading control.

Densitometry analysis was carried out by using ImageJ64, and intensities of bands were corrected to control lanes for each individual blot. All analyses were carried out on at least three individual repeats of the experiment.

### Acini assay

10A cont and 10A L2 cells were plated on top of a thin layer of 50-μl Matrigel in eight-well chamber slides, at a density of 10,000 cells/ml in 400 μl of 2% Growth Factor Reduced Matrigel without phenol red (BD Biosciences) in DMEM/F-12 media supplemented with 2% HS, 0.5 μg/ml hydrocortisone, 10 μg/ml insulin, 100 ng/ml cholera toxin, and 20 ng/ml EGF (Gibco/Invitrogen). The cultures were allowed to grow for 8, 10, and 13 days, and then fixed with 2% paraformaldehyde (PFA, Invitrogen) in PBS at room temperature.

For drug-treated cultures, lapatinib dissolved in 0.1% DMSO or Herceptin dissolved in PBS was added to cells at day 6 (10A cont+lap, 10A L2+lap; 10A cont+her, 10A L2+her), and 0.1% DMSO or an equivalent amount of human IgG was added as a control to lapatinib and Herceptin, respectively (10A cont+DMSO, 10A L2+DMSO; 10A cont+her, 10A L2+her). All assay media, with or without treatments, were changed every 3 to 4 days.

Acini were permeabilized with 0.5% Triton X-100 (Invitrogen), washed thoroughly with glycine buffer (0.1 *M *NaCl, 0.1 *M *glycine in PBS), blocked in IF buffer (0.1% bovine serum albumin (BSA), 0.2% Triton X-100, 0.05% Tween20, 130 m*M *NaCl in PBS) containing 10% goat serum (Invitrogen) and incubated with primary antibodies diluted in blocking buffer. Samples were then washed with IF buffer and incubated with appropriate Alexa-conjugated secondary antibodies (Invitrogen) in 10% goat serum/IF buffer. Nuclei were counterstained with DAPI (Invitrogen). Slides were then mounted under coverslips with Fluorescent Mounting Medium (Invitrogen) and visualized with confocal microscopy (Zeiss LSM710) by using a 63 × oil objective. Primary antibodies used were Ki-67, activated caspase-3, GM130, E-cadherin, and laminin V (all from Abcam). All photos taken were of midsections of acini. Photos presented here are representative of the overall phenotype in each experimental condition; quantifications were carried out on at least 20 acini per experimental repeat.

### Transwell invasion assay

Matrigel transwell invasion assays were carried out by using BD Bio-coat Matrigel Invasion Chambers (BD Biosciences), according to the manufacturer's instructions. Cells were serum-starved overnight before plating, and the normal culture media were added to the bottom chambers as chemoattractant. Plates were incubated at normal culture conditions for 24 hours before noninvading cells were removed from the inside of inserts, and membranes fixed and stained. Total number of invaded cells was counted under a light microscope (Nikon Diaphot 300).

### Branching assay

The 350,000 cells were plated on Matrigel-coated six-well plates. For treated cells, lapatinib (DMSO as control) or Herceptin (human kappa IgG as control) were added to cell cultures 3 hours after plating. Cells were then incubated overnight and viewed under a microscope (Leica DM1L).

### Soft agar assay

To test anchorage-independent growth in soft agar, 3 × 10^4 ^cells were suspended in 2 ml of 0.4% low-melting-point agarose (Invitrogen) in culture medium and were overlaid over 2 ml of 0.8% low-melting-point agarose in culture medium in a six-well plate. Culture medium was added to the top once the agarose had solidified, and plates were incubated at 37°C, 5% CO_2_/21% O_2_. After 3 weeks in culture, colonies were stained with 0.1% crystal violet and destained with PBS. Colonies with more than 20 cells were scored with a light microscope (Nikon Diaphot 300).

### Activity assay

Activity assays using a fluorescence-based LOX family enzymatic-activity assay measuring H_2_O_2 _(Abcam, Ab112139) were carried out according to manufacturer's instructions except using Matrigel as the substrate. Then 5 μl of concentrated conditioned media was added to the reaction mixture on a thin layer of Matrigel in a 96-well dish.

### Patient data analysis

Gene-expression microarray data from a previously published NKI cohort [17] was first transformed by using Disease Specific Genomic Analysis (DSGA) [18] to identify the extent of deviation from a Healthy State Model (HSM), defined by 13 normal breast samples [18]. This provided gene-expression levels in tumors that are relative to the control normal tissue data. Of 295 tumors, 51 were found to express high levels of ErbB2, and a Kaplan-Meier survival curve is constructed from this subset of ErbB2-positive tumors. Shown here is the overall and metastasis-free survival of patients with Her2/ErbB2-positive tumors (*n *= 51) separated into low and high LOXL2 expression.

Validation was carried out by combining another two previously published NKI cohorts [19,20]. Samples were run on Affymetrix U133a chips according to manufacturer's instructions, and data were obtained by using MAS5 parameters with global scaling set to 600. Of 344 tumors, 88 were found to express high levels of ErbB2, and Kaplan-Meier survival curves were constructed from this subset of ErbB2-positive tumors. Shown here is the overall and metastasis-free survival of patients with Her2/ErbB2-positive tumors (*n *= 88) separated into low and high LOXL2 expression.

### Statistical analysis

Data were analysed by using the Student *t *test, and considered significant at *P *≤ 0.05. All statistical tests were two sided. Bar graphs represent the mean and standard error across independent experimental repeats, and all experiments were performed at least three independent times. Statistical significance representations: *P < 0.05; **P < 0.01; ***P < 0.001; and ****P < 0.0001.

## Results and Discussion

### Expression of LOXL2 in human MCF10A normal mammary epithelial cells

We first investigated the potential impact of LOXL2 expression in normal breast epithelial morphogenesis. The MCF10A cell line was derived from normal human mammary epithelium that had undergone spontaneous immortalization without transformation, and is not tumorigenic [21]. With this normal epithelial cell line, we investigated how LOXL2 promotes tumor progression. We infected MCF10A cells with pBABE retroviral vector containing a *LOXL2 *cDNA to generate LOXL2-expressing cells (10A L2) and generated control cells (10A cont) by infecting wild-type MCF10A cells with the parental pBABE retroviral vector. MCF10A cells are known to show genetic drift [22]; however, we maintained cells at a passage lower than 20, as we noted altered cell phenotype above passage 30, consistent with a previous report [22].

**Figure 1:**
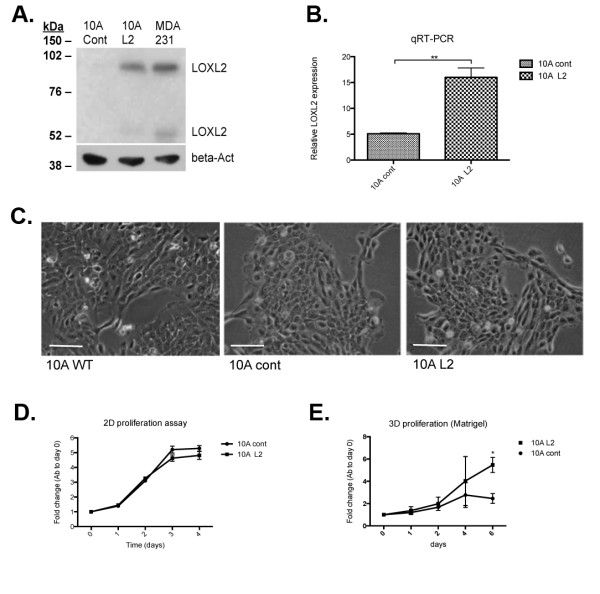
**Manipulation of LOXL2 expression levels in human MCF10A normal mammary epithelial cells**. **(A) **Western blot of secreted LOXL2 in CM generated from MDA MB-231 cells (MDA 231), MCF10A cells infected with LOXL2 (10A L2) and vector alone (10A cont) revealed that LOXL2 protein expression was upregulated in the 10A L2 cells to a level similar to that detected in MDA cells. β-Actin was used as a loading control. (B) Quantitative real-time PCR (qRT-PCR) of *LOXL2 *mRNA levels in manipulated MCF10A cells showed that *LOXL2 *mRNA levels were upregulated in 10A L2 cells. *P *= 0.009. (C) Morphologies of the manipulated MCF10A cells compared with WT cells showed that upregulation of LOXL2 did not produce significant alterations on cells plated on 2D tissue-culture plastic. Cells were viewed under a microscope (Leica DM1L), and representative images were taken. (D) 2D MTS proliferation assay of manipulated MCF10A cells suggested that when cultured on plastic, LOXL2 expression did not alter proliferation of the 10A cells. Error bars represent SEM for three independent experiments. (E) 3D MTS proliferation assay of manipulated MCF10A cells with manipulated LOXL2 expression plated within Matrigel suspension suggested that increased LOXL2 expression increases proliferation of the 10A cells in 3D. Error bars represent SEM for three independent experiments. *P *= 0.034 for day 6.

Western blot analysis showed that secreted LOXL2 was upregulated in 10A L2 cells relative to control cells, and comparable with those in the human breast cancer cell line MDA-MB-231 (Figure 1A). Two bands of approximately 95 kDa and 65 kDa were observed, the 95-kDa band representing the long form of LOXL2 that is commonly reported in cancer cells, and the fainter 65-kDa band that may correspond to the proteolytically modified short form of LOXL2 protein [9,23]. Quantitative real-time PCR indicated that *LOXL2 *mRNA expression was significantly increased in 10A L2 cells (*P *< 0.05; Figure 1B).

Previous reports suggested that manipulated LOXL2 expression in normal mammary epithelial cells [24] and MDA-MB-231 human breast cancer cells [25] can alter cell morphology on 2D tissue-culture plastic. However, we did not observe any significant phenotypic alterations when compared with 10A wild-type cells at passage 3 (Figure 1C), consistent with our previous findings with MDA-MB-231 and 4T1 cells, in which no obvious changes in 2D morphology were noted [4]. These findings suggest variable effects of LOXL2 on 2D morphology that may be cell-line specific or sensitive to cell-culture methods.

To assess the effects of LOXL2 on 2D proliferation, we carried out MTS assays. Increased secretion of LOXL2 did not affect 2D cell proliferation on plastic (Figure 1D). In contrast, we observed a significant increase in proliferation of 10A L2 cells compared with 10A cont cells after 6 days of culture in Matrigel suspension (*P *< 0.05; Figure 1E). Taken together, these results suggest that the microenvironment is important for the effects of LOXL2.

### LOXL2 expression disrupts normal breast epithelial acini formation

**Figure 2:**
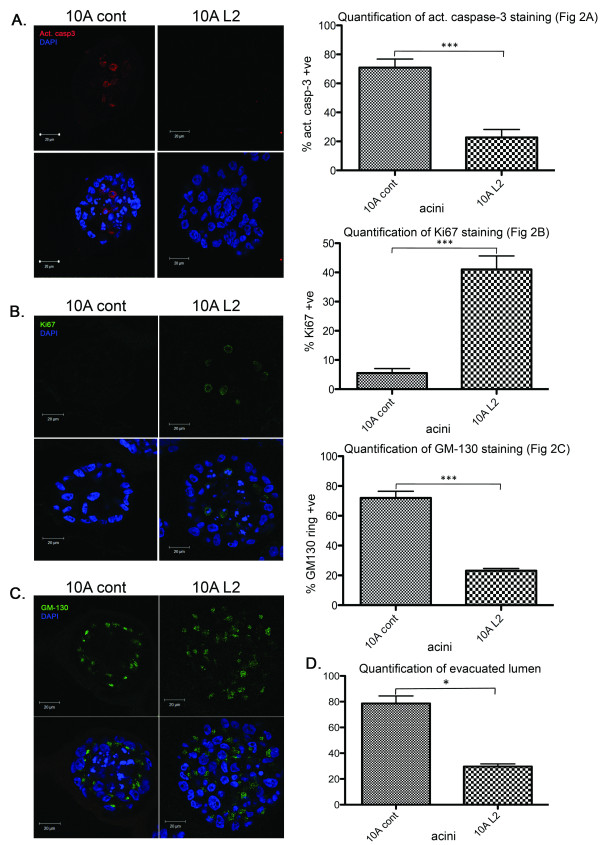
**LOXL2 expression disrupts normal breast epithelial acini formation**. 10A cont and 10A L2 cells were plated on top of a thin layer of 50 μl Matrigel in eight-well chamber slides in a suspension of Matrigel/culture media mix to investigate the acinar morphogenesis of these cells. The cultures were allowed to grow for 8, 10, and 13 days, and then fixed and stained with primary antibodies, as described in the different panels. All data were based on at least three independent experimental repeats. Scale bar, 20 μm. (A) Immunofluorescence staining of acini with anti-activated caspase-3 antibody to detect apoptotic cells on day 8 reveals decreased apoptosis in the central cells of the L2 acini. Representative images of activated caspase-3 staining in acini for each cell line are shown. Quantification represented average percentage ± standard error of acini containing activated caspase-3-positive cells (*P *= 0.0007). (B) Immunofluorescence staining of acini with anti-Ki67 antibody to detect proliferating cells on day 13 reveals that L2 acini had more proliferative cells. Representative images of Ki67-positive acini from each cell line are shown. Quantification represented average percentage ± standard error of acini containing Ki67-positive cells. (*P *= 0.0007). (C) Immunofluorescence staining of acini with anti-GM130 antibody to assess cell polarity on day 10. Representative images of GM130 staining in acini for each cell line are shown. Quantification represented average percentage ± standard error of acini forming a regular ring structure, as assessed by GM130 staining (*P *= 0.0005; *n *= 80 acini for each cell line per repeat). (D) Quantification of average percentage of acini at day 13 with evacuated lumens ± standard error. Acini with evacuated lumens were defined as having no more than 20% of total number of cells, as well as Ki67-positive cells present in the center (*P *= 0.013).

The role of LOXL2 in 3D development of normal breast epithelial cells was assessed by using the well-characterized MCF10A acini assay [14], in which cells are cultured on top of a layer of recombinant basement membrane (Matrigel) within a Matrigel suspension. The 10A cont and 10A L2 cells were compared for their ability to form acini structures in Matrigel over a 13-day period. In 10A cont acini, lumen formation was initiated by apoptosis, as assessed by the expression of activated caspase-3, in the middle of the acinar structure at day 8, as expected. In contrast, significantly fewer central cells in 10A L2 acinar structures expressed activated caspase-3 (*P *< 0.001; Figure 2A). Consistent with our finding, it was previously reported that genetic silencing of *LOXL2 *increased apoptosis in carcinoma tumor growth [26].

During normal acini structure development, proliferation ceases after 12 days of culture when lumen formation is complete [14]. In this study, we investigated the effect of LOXL2 overexpression on proliferation through Ki67 immunofluorescence staining. After 13 days in culture, control acini formed lumens and had significantly fewer proliferating cells, whereas proliferating cells were still present in 10A L2 acini (*P *< 0.001; Figure 2B). This sustained proliferation observed may explain the larger size of the 10A L2 acini. Moreover, 10A L2 acini did not have fully evacuated lumens (Figure 2B and 2D), similar to acini responding to oncogene activation [27]. The increased proliferation was consistent with the growth of the cells in Matrigel (Figure 1E). These results stress the importance of the surrounding microenvironment for studying cellular processes in culture [28], especially those involved in cancer progression [15,29].

Mammary epithelial polarization is an important process during acini formation. One of the characteristics of polarization is the redistribution of cellular organelles. The Golgi apparatus, in particular, is aligned on the apical side of polarized epithelial cells [30]. Immunofluorescence staining of 10A cont acini for GM130, a Golgi marker, revealed that by day 10 of culture, the Golgi apparatus in the outer cells of the acini was aligned toward the lumen, forming a classic ring-like pattern, as previously described [31,32]. In contrast, cells in the 10A L2 acini failed to polarize, as evidenced by the disorganized GM130 staining (*P *< 0.001; Figure 2C). It was recently proposed that epithelial cell polarity may play a major role in tumor initiation and progression [33], and that LOXL2 decreases the expression of cell-polarity complexes in basal-like breast carcinoma cells [34]. This provides a link between our previous report of LOXL2 mediating breast cancer progression [4], and our current report that LOXL2 expression drives abnormal cell polarity in normal mammary epithelial cells.

As acinar lumen filling is a characteristic of oncogenic transformation [35], we next investigated whether overexpression of LOXL2 alone can elicit oncogenic activity in the MCF10A cells. We carried out a soft agar assay for anchorage-independent cell growth. The metastatic human breast cancer cell line MDA-MB-231 was used as a positive control for colony formation in soft agar. After 3 weeks in culture, neither the 10A cont nor the 10A L2 cells proliferated in soft agar (Supplementary Figure 1), suggesting that LOXL2 expression alone cannot drive complete oncogenic transformation in the normal 10A cells.

These findings indicate that increased LOXL2 expression in normal mammary epithelial cells sustains proliferation, represses apoptosis, and disrupts polarization. These are all hallmarks of cancer cells [36], suggesting that aberrant expression of LOXL2 may drive normal mammary epithelial cells to behave like cancer cells; however, LOXL2 alone is not sufficient to drive complete oncogenic transformation.

### LOXL2 expression activates ErbB2 signaling

**Figure 3:**
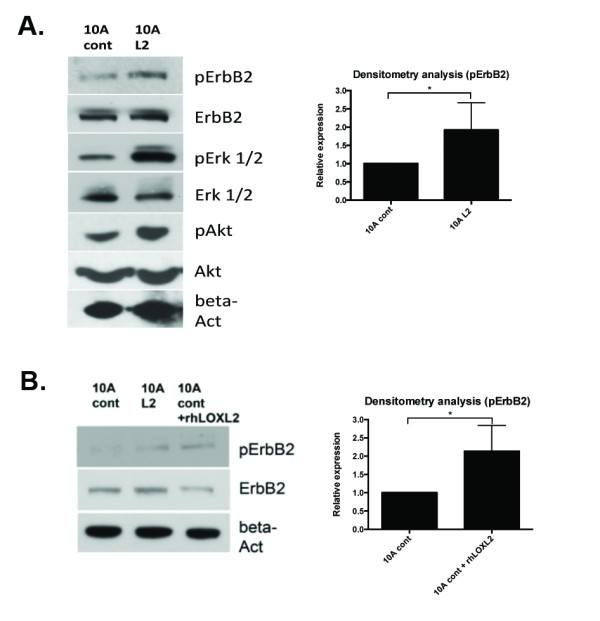
**LOXL2 expression in MCF10A cells increases phosphorylation of ErbB2**. Cells were plated on a thin layer of Matrigel and serum-starved for 3 hours before being subjected to serum-blasting and subsequent lysis of cells. (A) Western blotting revealed that phospho-ErbB2 was elevated in 10A L2 cells when compared with 10A cont cells, whereas total ErbB2 levels were equivalent in the two lines, suggesting increased phosphorylation of ErbB2 in the 10A L2 cells. Densitometry analysis was calculated for pErbB2 levels relative to total ErbB2 and revealed a significant increase in 10A L2 cells (*P *= 0.0241). The levels of phospho-Akt and phospho-Erk1/2 were also elevated in 10A L2 cells. (B) The 10A cont cells were subjected to 16-hour treatment with 50 nM recombinant human LOXL2 (rhLOXL2; R&D Systems) followed by serum-starvation and serum-blasting (10A cont + rhLOXL2). Western-blotting analysis showed that in 10A cont treated with rhLOXL2, phospho-ErbB2 level was increased to a greater extent than 10A L2 cells when compared with sham-treated 10A cont cells. Densitometry analysis was calculated for pErbB2 levels relative to total ErbB2 and revealed significant increase in 10A cont + rhLOXL2 cells (*P *= 0.0438). This suggested that extracellular recombinant LOXL2 was capable of activating the ErbB2 receptor in 10A cells.

We noted the similarity between the morphology of LOXL2-expressing acini and ErbB2 activation in acini [35]. This led us to investigate whether LOXL2-mediated aberrant acini formation occurs through ErbB2 signaling. We performed Western blotting on cell lysates from cells plated on Matrigel-coated plates, and noted elevated phospho-ErbB2 in the 10A L2 cells compared with the 10A cont cells (Figure 3A). Consistently, we observed elevated phospho-Akt (PKB), a known downstream marker of ErbB2 activation [37,38], in the 10A L2 cells compared with 10A cont cells (Figure 3A). We also observed increased pErk1/2 levels in the 10A L2 cells, suggesting that the Ras-MAPK pathway was also activated. Previous reports have demonstrated the role of intracellular LOXL2 in cancer progression and cell polarity [25,39], and indeed, in our cells, the stable expression of LOXL2 increases intracellular LOXL2 levels in MCF10A cells (Supplementary Figure S2A). Thus the effects of LOXL2 on acinar morphology and ErbB2-related signaling events may be due to intracellular LOXL2.

To distinguish the effects of extracellular LOXL2 on these signaling events, we treated Matrigel-plated 10A cont cells with recombinant human LOXL2 (rhLOXL2). In this setting, we also observed elevated levels of phospho-ErbB2 (Figure 3B), indicating that extracellular LOXL2 can induce ErbB2 phosphorylation. The reduction in total ErbB2 after overnight rhLOXL2 treatment is likely the result of a feedback mechanism to compensate for increased signalling from the receptor due to the presence of rhLOXL2. This reduction was not observed in the 10A L2 cells and may thus be a result of the cells reaching homeostasis in terms of ErbB2 expression because of long-term exposure to LOXL2.

We used the ErbB2-specific inhibitor Herceptin (Her) to investigate the role of ErbB2/Her2 in mediating the observed effects of LOXL2 expression. It was previously reported that when used at a concentration of 338 n*M*, Herceptin kills 10% of MCF7 cells, a breast epithelial cancer cell line with low ErbB2 expression levels [40]. We observed no differences in the proliferation of 10A cont and L2 cells at this concentration (data not shown), consistent with the nonamplified ErbB2 status of the MCF10A cell line; thus we proceeded with a treatment concentration of 300 n*M *at day 6 of culture, with the equivalent amount of human IgG added as controls. Western blot analysis showed that phosphorylation of ErbB2 was inhibited at this concentration (Supplementary Figure S2B).

### Inhibition of ErbB2 restores the normal phenotype in LOXL2-expressing acini

**Figure 4:**
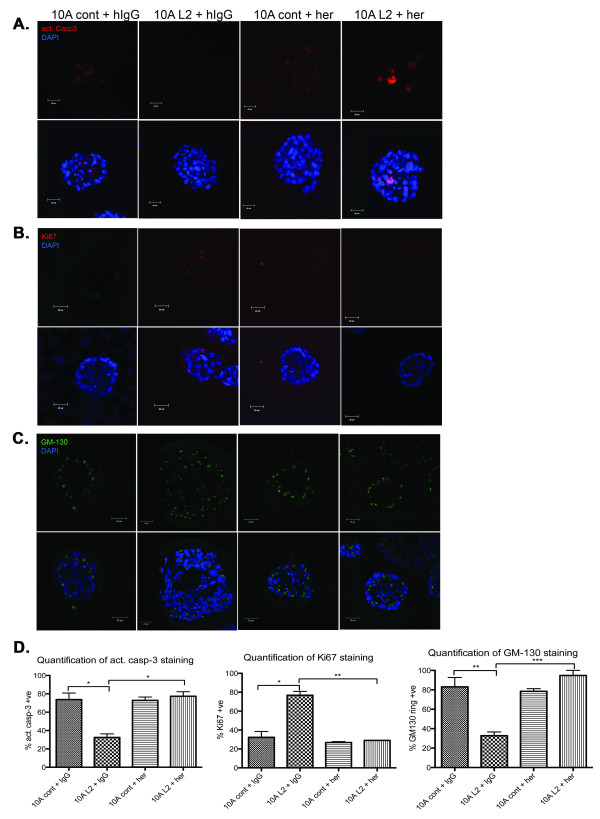
**Treatment with the ErbB2-specific inhibitor trastuzumab (Herceptin) restores LOXL2-expressing acini phenotype to a more normal phenotype**. Acini were cultured as described in Figure 2; 300 nM Herceptin (Her) dissolved in water was added to cells at day 6 (10A cont+her; 10A L2+her), and an equivalent amount of human IgG was added as a control (10A cont+hIgG; 10A L2+hIgG). Acini were fixed, stained, quantified, and presented as described in Figure 2. All data are based on at least three independent experimental repeats. Scale bar, 20 μm. (A) Immunofluorescence staining of acini with anti-activated caspase-3 antibody to detect apoptotic cells on day 8. Staining revealed increased activation of caspase-3 in 10A L2+her acini when compared with 10A L2+hIgG acini. (B)Immunofluorescence staining of acini with anti-Ki67 antibody to detect proliferating cells on day 13. Staining revealed a decrease in proliferating cells in 10A L2+her acini when compared with 10A L2+hIgG acini. (C) Immunofluorescence staining of acini with anti-GM130 antibody to assess cell polarity on day 10. Staining revealed GM-130 ring-like staining effect in 10A L2+her acini, compared with the scrambled and disorganized staining observed in 10A L2+hIgG acini. (D) Quantitative analysis of activated caspase-3 staining (left panel; *P *= 0.0364 for 10A cont+hIgG and 10A L2+hIgG; *P *= 0.0189 for 10A L2 ± her), Ki67 staining (middle panel; *P *= 0.0268 for 10A cont+hIgG and 10A L2+hIgG; *P *= 0.0073 for 10A L2 ± her), and GM130-ring structures (right panel; *P *= 0.0083 for 10A cont+IgG and 10A L2+IgG; *P *= 0.0007 for 10A L2 ± her) in acini. These results suggest that Herceptin treatment of 10A L2 acini significantly reverted the acinar morphology to a more-normal phenotype.

Herceptin was added on day 6 of acini culture, with equivalent dilution of human kappa IgG added to controls. IgG-treated 10A L2 acini showed a reduced level of apoptosis (Figure 4A), as observed in the untreated 10A L2 acini (Figure 2A). Inhibition of ErbB2 by using Herceptin significantly induced apoptosis in the central cells of these acini (*P *< 0.05; Figure 4D), to a level equivalent to that seen in IgG-treated 10A cont acini (Figure 4D, left panel). Apoptosis in 10A cont acini was unaffected by Herceptin treatment (Figure 4A).

Ki67 staining in 10A L2 acini treated with IgG remained high, as observed in untreated acini, whereas 10A L2 acini treated with Herceptin exhibited significantly decreased Ki67 expression (*P *< 0.01; Figure 4D), which was comparable to the 10A cont acini (Figure 4B).

The alignment of the Golgi apparatus in 10A L2 acini was also restored in response to Herceptin treatment, such that a classic ring-like staining pattern similar to control acini was observed (Figure 4C). In contrast, the Golgi apparatus in IgG-treated 10A L2 acini retained the scrambled and disorganized staining pattern observed in untreated 10A L2 acini. Quantification revealed that the polarity of 10A L2 acini treated with Herceptin was comparable to that of control acini (*P *< 0.005, Figure 4D), and 10A cont acini were unaffected by Herceptin treatment (Figure 4D, right panel). These findings suggest that inhibition of ErbB2 blocks the effects of LOXL2 on mammary epithelial cell polarization. In this model, the 10A L2 acini appear more responsive to Herceptin than 10A cont acini, likely because of the constitutive high levels of ErbB2 activation present in these cells driven by elevated levels of LOXL2, whereas 10A cont acini do not have LOXL2-driven overactivation of ErbB2.

To verify that the differences observed in the acini after Herceptin treatment were indeed due to inhibition of ErbB2, we used lapatinib, a dual ErbB1/ErbB2 small-molecule inhibitor, as an additional treatment. It has been previously reported that in MCF10A cells, the IC_50 _concentration for lapatinib is 800 n*M *[41]. We observed no differences in the proliferation of 10A cont and L2 cells at this concentration (data not shown), and an effect on the 10A L2 cells is found only at 5 μ*M*. Western blot analysis revealed that 5 μ*M *was needed to inhibit ErbB2 phosphorylation in the 10A L2 cells (Supplementary Figure S2C). The elevated total ErbB2 levels are likely due to a feedback mechanism used by the cell to compensate for decreased receptor signaling.

**Figure 5:**
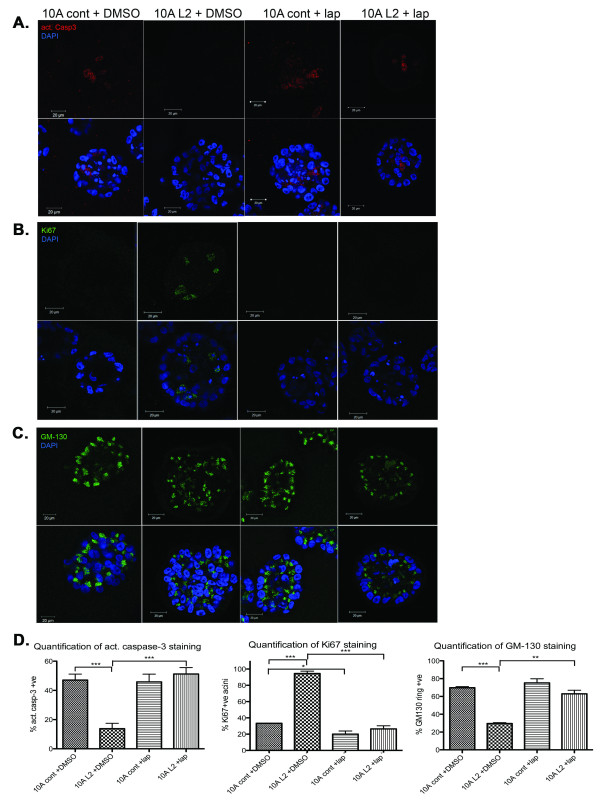
**The dual ErbB1/ErbB2 inhibitor lapatinib reverts LOXL2-mediated acinar morphologic changes to a more-normal phenotype**. Acini were cultured as described in Figure 2; 5 μM lapatinib (lap) dissolved in DMSO was added to cells at day 6 (10A cont+lap; 10A L2+lap), and equivalent amounts of DMSO were added as a control (10A cont+DMSO; 10A L2+DMSO). Acini were fixed, stained, and quantified as described. All data are based on at least three independent experimental repeats. Scale bar, 20 μm. (A) Immunofluorescence staining of acini with anti-activated caspase-3 antibody to detect apoptotic cells on day 8. Staining revealed increased activation of caspase-3 in 10A L2+lap acini when compared with 10A L2+DMSO acini. (B) Immunofluorescence staining of acini with anti-Ki67 antibody to detect proliferating cells on day 13. Staining revealed a decrease in proliferating cells in 10A L2+lap acini when compared with 10A L2+DMSO acini. (C) Immunofluorescence staining of acini with anti-GM130 antibody to assess cell polarity on day 10. Staining revealed GM-130 ring-like staining effect in 10A L2+lap acini, compared with the scrambled and disorganized staining observed in 10A L2+DMSO acini. (D) Quantitative analysis of activated caspase-3 staining (left panel; *P *= 0.00047 for 10A cont+DMSO and 10A L2+DMSO; *P *= 0.0006 for 10A L2 ± lap), Ki67 staining (middle panel; *P *= 0.00032 for 10A cont+DMSO and 10A L2+DMSO; *P *= 0.0257 for 10A cont ± lap; *P *= 0.00013 for 10A L2 ± lap), and GM130-ring structures (right panel. *P *= 0.00016 for 10A cont+DMSO and 10A L2+DMSO; *P *= 0.0013 for 10A L2 ± lap) in acini. These results suggest that lapatinib treatment of 10A L2 acini significantly reverted the acinar morphology to a more-normal phenotype.

Activated caspase-3, Ki67, and GM130 staining revealed that 10A L2 acini reverted to a more-normal phenotype when treated with lapatinib (Figure 5A through C, respectively), confirming our findings with Herceptin, and showing that inhibition of ErbB2 blocks LOXL2-mediated formation of aberrant mammary acini. Interestingly, a significant decrease in Ki67 staining (*P *< 0.05) was seen between DMSO-treated 10A cont acini and those treated with lapatinib, suggesting that lapatinib affects normal mammary epithelial cell proliferation (Figure 5D, middle panel). However, lapatinib has a greater efficacy on 10A L2 acini, consistent with higher levels of phospho-ErbB2 in the 10A L2 cells. Of interest, we noted a slight decrease in *LOXL2 *mRNA expression in response to ErbB2 inhibition (data not shown), consistent with a previous report [42], in which it was demonstrated that ErbB2 may regulate LOXL2 levels, suggesting the presence of a feed-forward mechanism.

We showed that Herceptin inhibited ErbB2 signaling in the 10A L2 cells, and reverted the aberrant 10A L2 acinar phenotype to a more-normal phenotype, suggesting that the LOXL2-induced effect on acinar morphogenesis was due to ErbB2 signaling. These results were verified by using lapatinib. Taken together, these findings show that blocking ErbB2 alone is sufficient to revert the disorganized morphology of 10A L2 acini to a normal morphology similar to that of 10A cont acini. The increase of pErk1/2 in the 10A L2 cells (see Figure 3A) is likely due to activation of ErbB2; it is known that phosphorylation of ErbB2 can be coupled to the Ras-MAPK pathway [43,44].

### Inhibition of ErbB2 prevents LOXL2-expressing cells from forming branched structures on Matrigel, and LOXL2 promotes invasion of normal breast epithelial cells

**Figure 6:**
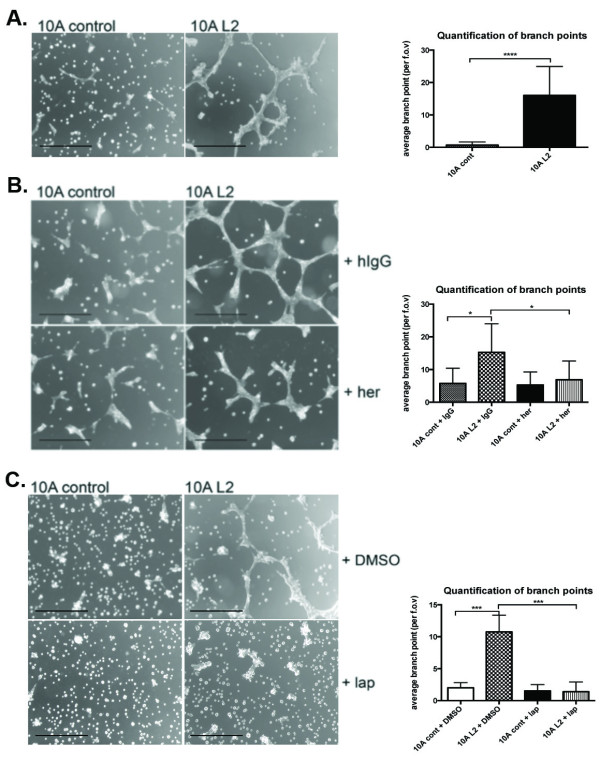
**LOXL2-expressing MCF10A cells form ErbB2-dependent branching structures on matrix**. (A) When plated on Matrigel-coated plates, 10A cont cells remained small and discrete, whereas 10A L2 cells formed extensive branching structures. Quantification of branch-points revealed that 10A L2 cells have significantly more branch-points (right panel; *P *= 0.00004). (B) Herceptin treatment of the 10A L2 cells significantly decreased the degree of branching of these cells to that seen for controls (right panel; *P *= 0.0167 and *P *= 0.0398, respectively). (C) Lapatinib treatment of the 10A L2 cells significantly abrogated the branching ability of these cells to that seen for controls (right panel; *P *= 0.0007 and *P *= 0.0003, respectively).

When plated on top of a thin layer of Matrigel (2D on matrix), 10A L2 cells formed branching structures, whereas 10A cont cells remained small and round (Figure 6A, left panel). We counted branch points as a quantification of branching structures, and 10A L2 cells showed significantly more branch points than did 10A cont cells (*P <*0.001; Figure 6A, right panel). For 10A cont cells, very little branching was observed in both hIgG- and Herceptin-treated samples, whereas Herceptin treatment of 10A L2 cells significantly decreased the extensive branching, and cells formed smaller and more discrete clusters, similar to the 10A cont cells (*P *< 0.05; Figure 6B). The extensive branching networks observed in 10A L2 cells were also abrogated by treatment with lapatinib (*P *< 0.001; Figure 6C). The slight decrease in 10A L2 branching as a result of DMSO treatment, as well as the slight induction of clustering of 10A cont cells under hIgG treatment, is likely due to nonspecific stress responses elicited by the cells toward these control treatments; however, these responses are minor in comparison to therapy treatment. These findings suggest that LOXL2 expression drives branching of MCF10A cells on Matrigel, and inhibition of ErbB2 abrogates this LOXL2-mediated effect.

**Figure 7:**
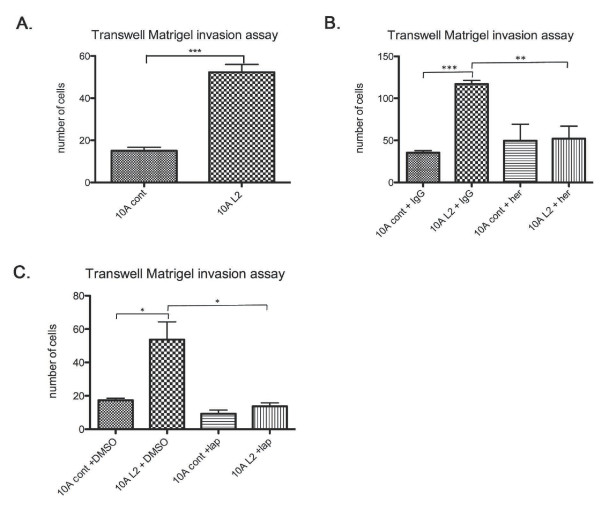
**LOXL2 promotes invasion of normal breast epithelial cells; this effect is abrogated with ErbB2 inhibition**. (A) The 10A L2 cells exhibited increased invasion through Matrigel compared with 10A cont cells in Transwell invasion assays. (B) Herceptin treatment reduced the invasive ability of the 10A L2 cells to a level comparable to 10A cont cells. Human IgG treatment had no effect on the invasiveness of the manipulated 10A cells. (C) Lapatinib treatment of the 10A L2 cells greatly reduced the invasiveness of the cells to a level comparable to the 10A cont cells. DMSO treatment had no effect on the invasive properties of the manipulated 10A cells.

As branching structures are indicative of an invasive phenotype [45,46], we investigated the invasive capabilities of these cells. Consistently, we observed that 10A L2 cells invaded more than control cells through Matrigel in Transwell invasion assays (Figure 7A). Moreover, the invasion of 10A L2 cells was significantly reduced by treatment with either Herceptin (Figure 7B) or lapatinib (Figure 7C). These findings are also in accordance with previous reports that ErbB2 activation induces invasion of MCF10A cells [47]. The branching morphology was not apparent in the 3D acini cultures, as confirmed by immunofluorescence staining of Laminin V, which indicated that the basement membrane was intact in both 10A L2 and 10A cont acini (Supplementary Figure S3A). It was previously reported that only acini with ErbB2/ErbB3 dual overexpression develop invasive structures, whereas ErbB2-only overexpressing acini have a larger acinar structure with a filled-in lumen [48]. The branching morphology present in 10A L2 cells in 2D culture and lack thereof in the 3D culture suggests that further signals, such as ErbB3 activation reported by Aceto *et al*. [48], may be required for the 3D branching to occur.

The presence of branching structures, as well as increased invasiveness when plated on top of Matrigel, suggests that the 10A L2 cells have undergone EMT [49]. Intracellular LOXL2 has previously been implicated in mediating EMT [8,26]. Thus, we investigated the mRNA levels of the EMT markers vimentin, E-cadherin, N-cadherin, SNAI1, SNAI2, Twist, and α-SMA in 10A cells plated on top of Matrigel. We found that only E-cadherin and SNAI1 were significantly downregulated at the mRNA level in the 10A L2 cells (Supplementary Figure S3B), suggesting that they may have lost some of their epithelial features. However, when we assessed E-cadherin expression in the 3D acinar structures through immunofluorescence staining, we found that E-cadherin protein levels were not altered between the two cell lines (Supplementary Figure S3C). When taken together, these data suggest that, when plated on matrix in a 2D system, the 10A L2 cells can undergo only partial EMT, as no mesenchymal markers investigated here were upregulated. Furthermore, when cultured in 3D Matrigel suspension, the 10A L2 cells are unable to maintain this partial EMT phenotype. This suggests that EMT does not play a role in LOXL2-induced invasiveness in our model system.

To assess the role of LOXL2 in invasion of ErbB2-expressing cancer cells and validate our findings in a second model, we used the noninvasive ErbB2-overexpressing cancer cell line MDA-MB-361. Transwell-invasion assays revealed that these cells exhibited increased invasion through ECM when treated with rhLOXL2 (Supplementary Figure S3D). We also carried out a 4-day 3D culture of these cells, as described by Kenny *et al*., [50], and observed that both MDA-MB-361 control and rhLOXL2-treated cells form individual cell clusters, as previously described [50]; thus LOXL2 cannot induce invasive "stellate" structures or branching morphologies in 3D (data not shown), consistent with the MCF10A data. These findings support the theory that further signals are required, in addition to LOXL2 expression, for 3D invasive structures to form.

Taken together, these results suggest that LOXL2 expression can promote the development of a more-aggressive cancer-like phenotype in normal mammary epithelial cells, through ErbB2 activation.

### LOXL2 activates ErbB2 via reactive oxygen species production

Next, we investigated how LOXL2 might be mediating activation of ErbB2. Jung *et al*. [51] recently demonstrated that TIMP-1 induces aberrant acinar morphogenesis in MDCK cells [51]. As we previously showed a strong link between LOXL2 and TIMP-1 in breast cancer cell invasion [4], we postulated that TIMP-1 may be playing a role in the aberrant morphogenesis in our system. However, TIMP-1 protein levels were unchanged between the 10A cont and L2 lines (Supplementary S3E), suggesting that TIMP-1 is not involved.

It also was previously demonstrated that increased matrix stiffness enhances invasiveness of ErbB2-transformed MCF10A cells [52]. We therefore investigated whether stiffness plays a role in LOXL2-mediated ErbB2 activation. However, in our model system, increasing matrix stiffness did not increase ErbB2 activation (data not shown). Thus, activation of ErbB2 could not be attributed to regulation of TIMP1 or to altered matrix stiffness.

**Figure 8:**
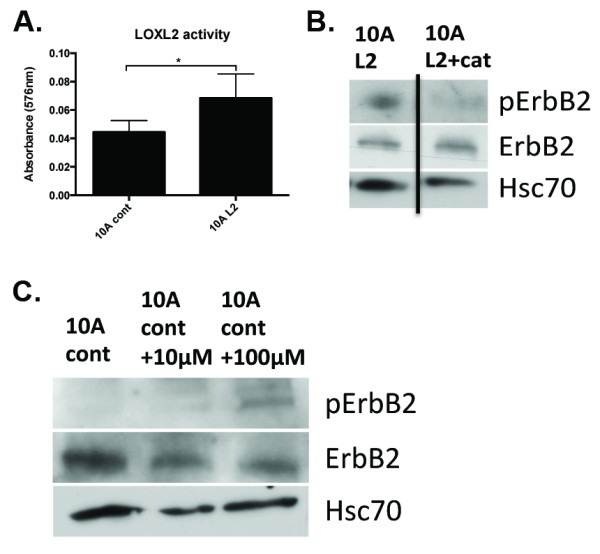
**LOXL2 expression induces ErbB2 activation through production of _2_O_2_**. (A)Production of H_2_O_2 _on Matrigel was measured as an activity read-out in the 10A cont and 10A L2 cells, by using a commercially available kit and following the manufacturer's instructions (Abcam). 10A L2 cells produced higher levels of H_2_O_2_, as expected, because of LOXL2 overexpression. (B) The 200 U/ml catalase (+cat) was added to remove H_2_O_2 _produced by LOXL2. Western blotting revealed that pErbB2 was decreased in 10A L2 cells treated with catalase (L2+cat) in comparison with untreated cells (L2). (C) Addition of H_2_O_2 _to 10A cont cells strongly induced ErbB2 activation. Taken together, these results indicate that, by removing H_2_O_2_, we can significantly abrogate ErbB2 activation in 10A L2 cells, and addition of H_2_O_2 _activated ErbB2.

We observed that LOXL2 activation of ErbB2 occurs within 15 minutes of stimulation (Supplementary Figure S4A), suggesting a novel mechanism of activation involving rapid receptor activation. It was previously shown that UV can rapidly activate ErbB2 through generation of reactive oxygen species (ROS) [53,54]. As such, we hypothesized that LOXL2 may activate ErbB2 through H_2_O_2 _produced as a byproduct of LOXL2 activity. The activity of LOXL2 is routinely assessed in the form of H_2_O_2 _production [55]. Indeed, we found CM from 10A L2 to have higher production of H_2_O_2 _compared with that from 10A cont cells (Figure 8A). The activity was measured in CM added on top of Matrigel, suggesting that Matrigel is acting as a substrate for LOXL2. We used catalase, an enzyme that removes H_2_O_2 _[56], to investigate the effects of H_2_O_2 _removal on ErbB2 activation. When 200 U/ml catalase was added to 10A L2 cells, we observed a significant reduction in ErbB2 activation (Figure 8B and Supplementary Figure S4B) without altering cell viability (data not shown). We confirmed these findings in 10A cont cells treated with rhLOXL2 for 15 minutes in the presence or absence of catalase (Supplementary Figure S4C). Moreover, addition of H_2_O_2 _to 10A cont cells was able to induce ErbB2 activation, confirming this as the likely mechanism of action (Figure 8C). Taken together, these results suggest that LOXL2 production of ROS can induce ErbB2 activation.

### LOXL2 expression correlates with metastasis and decreased survival in ErbB2-overexpressing breast cancer patients

**Figure 9:**
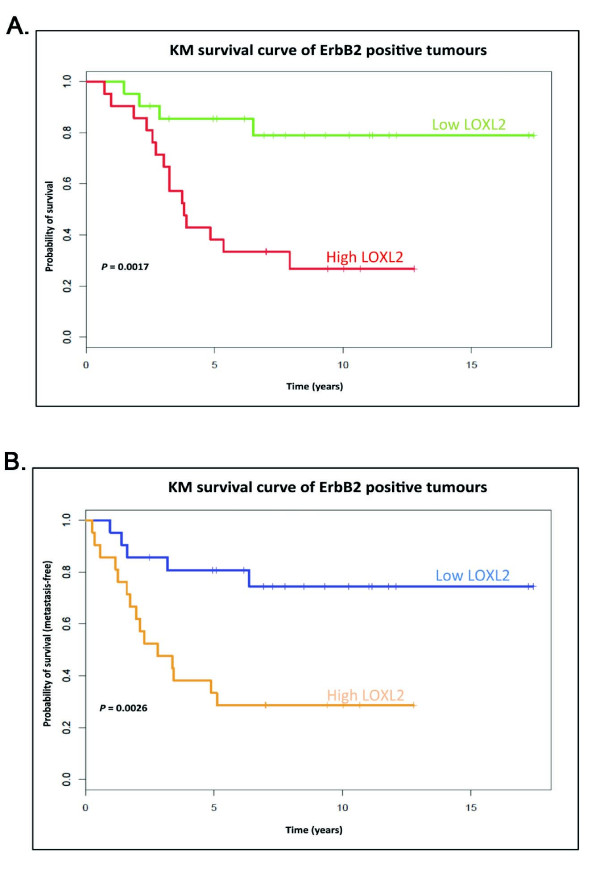
**Elevated LOXL2 and ErbB2 are associated with invasive cell behavior and poor patient prognosis**. (A) Disease-specific genomic analysis (DSGA) was performed on gene-expression microarray data from the NKI cohort [17]. Of 295 tumors, 51 were found to express high levels of ErbB2, and a Kaplan-Meier survival curve is constructed from this subset of ErbB2-positive tumors. Shown here is the overall survival of patients with Her2/ErbB2-positive tumors (*n *= 51) separated into low and high LOXL2 expression. *P *= 0.017. (B) As in Figure 8A, showing metastasis-free survival. *P *= 0.026.

Further to investigate the link between LOXL2 and ErbB2 and cancer progression in a clinical setting, we used a previously published dataset and performed a retrospective study to investigate whether *LOXL2 *mRNA expression level is correlated with metastasis and survival of ErbB2-positive breast cancer patients [17]. We found that patients with ErbB2-positive tumors expressing high levels of LOXL2 had a significantly poorer prognosis, with LOXL2 expression being significantly correlated with decreased overall survival and decreased metastasis-free survival in these patients (both *P *< 0.05; Figure 9A, B, respectively). The relation between LOXL2, ErbB2, and metastasis was further validated in a second patient data set (*P *≤ 0.05; Supplementary Figure S5).

We previously reported that LOXL2 expression in patients with ER^- ^tumors also correlates with metastasis and decreased survival [4]. Thus, LOXL2 expression is associated with poor prognosis in breast cancer patients with different subtypes of the disease. This is consistent with our observations that LOXL2 increases invasion and metastasis through the ErbB2-dependent mechanisms we present here, and through ErbB2-independent mechanisms reported previously [4,7].

## Conclusion

We showed that, in the immortalized human mammary epithelial cell line MCF10A increased expression of LOXL2 interferes with normal acini development by repressing apoptosis, prolonging proliferation, and abrogating polarization of the cells. We further showed that LOXL2 expression drives the formation of branching structures on matrix and enhances MCF10A cell invasion. We also demonstrated that ErbB2 is responsible for mediating LOXL2 effects on cell morphology and behavior, and present here an exciting new mechanism by which LOXL2 may regulate cellular behavior through production of ROS. Finally, we showed that high LOXL2 expression correlates with poor prognosis in ErbB2-positive breast cancer patients, demonstrating a strong link between LOXL2 and ErbB2 and aggressive cell behavior. Our findings indicate that high expression of LOXL2 promotes tumor-like phenotype in normal mammary cells and suggest that LOXL2 may be used as a marker to identify patients most likely to respond to anti-ErbB2 therapy. In addition, deactivation of ErbB2 may be used as a marker of response in patients treated with anti-LOXL2 therapy. Thus, our findings may have important clinical implications for breast cancer patients.

## Abbreviations

CM: conditioned media; DMSO: dimethyl sulfoxide; DSGA: disease-specific genomic analysis; EGF: epidermal growth factor; ErbB2: human epidermal growth factor receptor 2; GM130: 130-kDa cis-Golgi matrix protein; Her: Herceptin; HSM: healthy-state model; Ki67: antigen Ki-67; Lap: lapatinib; LOXL2: lysyl oxidase-like 2; LOX: lysyl oxidase; 10A cont, MCF10A control cell; 10A L2: MCF10A LOXL2 overexpressing cell; ErbB3: Receptor tyrosine-protein kinase erbB-3; TIMP-1: tissue inhibitor of metalloproteinase-1.

## Competing interests

The authors declare that they have no competing interests.

## Authors' contributions

JC, HEB, and JTE conceived of and designed the study. JC performed the *in vitro *experiments and drafted the manuscript. TRC, HEB, and JTE edited and finalized the manuscript. HEB generated the two MCF10A cell lines. MMN performed the retrospective DSGA, as well as interpretation of the data from the NKI patient cohort. JWM validated a second patient dataset. DW performed and analyzed IC_50 _experiments for the drugs used in this study. TRC assisted with matrix studies and interpretation of the data. All authors read and approved the final manuscript.

## Supplementary Material

Additional file 1 **Supplementary Figure S1**. **LOXL2 expression does not induce oncogenic activity**. The manipulated MCF10A cell lines were compared with MDA-MB-231 breast cancer cell line for their ability to grow in an anchorage-independent manner by using the soft agar assay. Results indicated that neither of the 10A cell lines can form colonies in soft agar, unlike the breast cancer cell line MDA-MB-231 (*P *= 0.00008). Click here for file

Additional file 2 **Supplementary Figure S2**. **LOXL2 expression and signaling inhibition in MCF10A cells**. (A) Western blot analysis of cell lysates from 10A cont and 10A L2 cells revealed that intracellular LOXL2 was also increased in the 10A L2 cells. (B) 10A L2 cells were subjected to overnight treatment with either 300 n*M *trastuzumab (Herceptin; L2+her) or the equivalent amount of human IgG (L2+IgG) followed by 3 hours of serum-starvation and 15 minutes of serum-blasting. Western blot analysis of the cell lysates revealed that at 300 n*M*, Herceptin inhibits the phosphorylation of ErbB2 in the 10A L2 cells. (C) 10A L2 cells were subjected to overnight treatment at the indicated dosage of lapatinib (0 μ*M *(that is, DMSO only), 1 μ*M*, and 5 μ*M*) followed by 3 hours of serum-starvation and 15 minutes of serum-blasting. Western blot analysis indicated that lapatinib inhibits phosphorylation of ErbB2 at only 5 μ*M *concentration. Click here for file

Additional file 3 **Supplementary Figure S3**. **LOXL2 does not induce invasion or EMT in 10A acini, but increases invasiveness of MDA-MB-361 cells. TIMP-1 levels are not altered by LOXL2 expression**. (A) Acini were cultured as described in Figure 2 and then fixed and stained with laminin V (Lam-V; Millipore) for the basement membrane and Ki-67. Representative photos from three independent experimental repeats are presented. Scale bar, 20 μm. Intact basement membrane, as evidenced by Lam-V staining, suggested that these cells do not form invasive structures when cultured in 3D. (B) Quantitative RT-PCR of *Vimentin*, *E-cadherin, N-cadherin, SNAI1, SNAI2, Twist*, and *α-SMA *in manipulated MCF10A cells showed that only *E-cadherin *and *SNAI1 *mRNA levels were downregulated in 10A L2 cells. Error bars represent SEM of three independent experiments. *P *= 0.00005 for E-cadherin and *P *= 0.00039 for *SNAI1*. (C) Acini were cultured as described in Figure 2 and then fixed and stained with E-cadherin (E-cad, Abcam). Representative photos from three independent experimental repeats are presented. Scale bar, 20 μm. 10A L2 acini did not have decreased E-cadherin protein levels. (D) The noninvasive ErbB2-amplified breast cancer MDA-MB-361 cells were subjected to Transwell invasion assays in the presence or absence of 50 n*M *rhLOXL2. Results indicated that recombinant LOXL2 increased the invasiveness of the noninvasive cells. *P *= 0.0176. (E) Western blot analysis of TIMP-1 levels in 10A cont and 10 L2 CM. Results indicate that TIMP-1 levels are unchanged between the two cell lines. Click here for file

Additional file 4 **Supplementary Figure S4**. **Recombinant human LOXL2 (rhLOXL2) rapidly activates ErbB2, and H_2_O_2 _depletion inhibits this activation**. (A) The 10A cont cells were plated out, as described in Figure 3. After overnight incubation, cells were serum-starved for 3 hours and stimulated with 50 n*M *rhLOXL2 for 15 minutes. Western blot analysis revealed that ErbB2 was activated rapidly. Densitometry analysis was calculated on pErbB2 expression relative to Hsc70. (B) Densitometry analysis revealed that catalase treatment significantly decreased activation of ErbB2 in 10A L2 cells (*P *= 0.026) and was calculated on pErbB2 expression relative to ErbB2. (C) Catalase treatment (cont+rh+cat) abrogated rhLOXL2-mediated ErbB2 activation in 10A cont cells (cont+rh) (right blot, *P *= 0.0017 for cont versus cont+rhLOXL2; *P *= 0.043 for cont+rhLOXL2 versus cont+rhLOXL2+cat). Densitometry analysis was calculated on pErbB2 expression relative to Hsc70. Click here for file

Additional file 5 **Supplementary Figure S5**. **LOXL2 expression is correlated with metastasis in breast cancer patients with ErbB2^+ ^tumors**. Kaplan-Meier survival curves were constructed for patients with Her2/ErbB2-positive tumors by using MAS5 parameters, with global scaling set to 600 [19]. Overall survival (left panel; *P *= 0.05) and metastasis-free survival (right panel; *P *= 0.032) are shown here; *n *= 88 patients. Click here for file
