# Supplementary figures and images for: LOXL2 induces aberrant acinar morphogenesis via ErbB2 signaling
Source: Breast Cancer Res. 2013 Aug 23;15(4):R67. doi: 10.1186/bcr3461 (PMC3978831; doi:10.1186/bcr3461)

## Colonization in Soft Agar

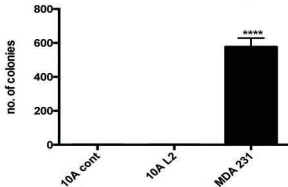

Supplement: Additional file 1 — Supplementary Figure S1. LOXL2 expression does not induce oncogenic activity. The manipulated MCF10A cell lines were compared with MDA-MB-231 breast cancer cell line for their ability to grow in an anchorage-independent manner by using the soft agar assay. Results indicated that neither of the 10A cell lines can form colonies in soft agar, unlike the breast cancer cell line MDA-MB-231 (P = 0.00008). [file bcr3461-S1.PDF]

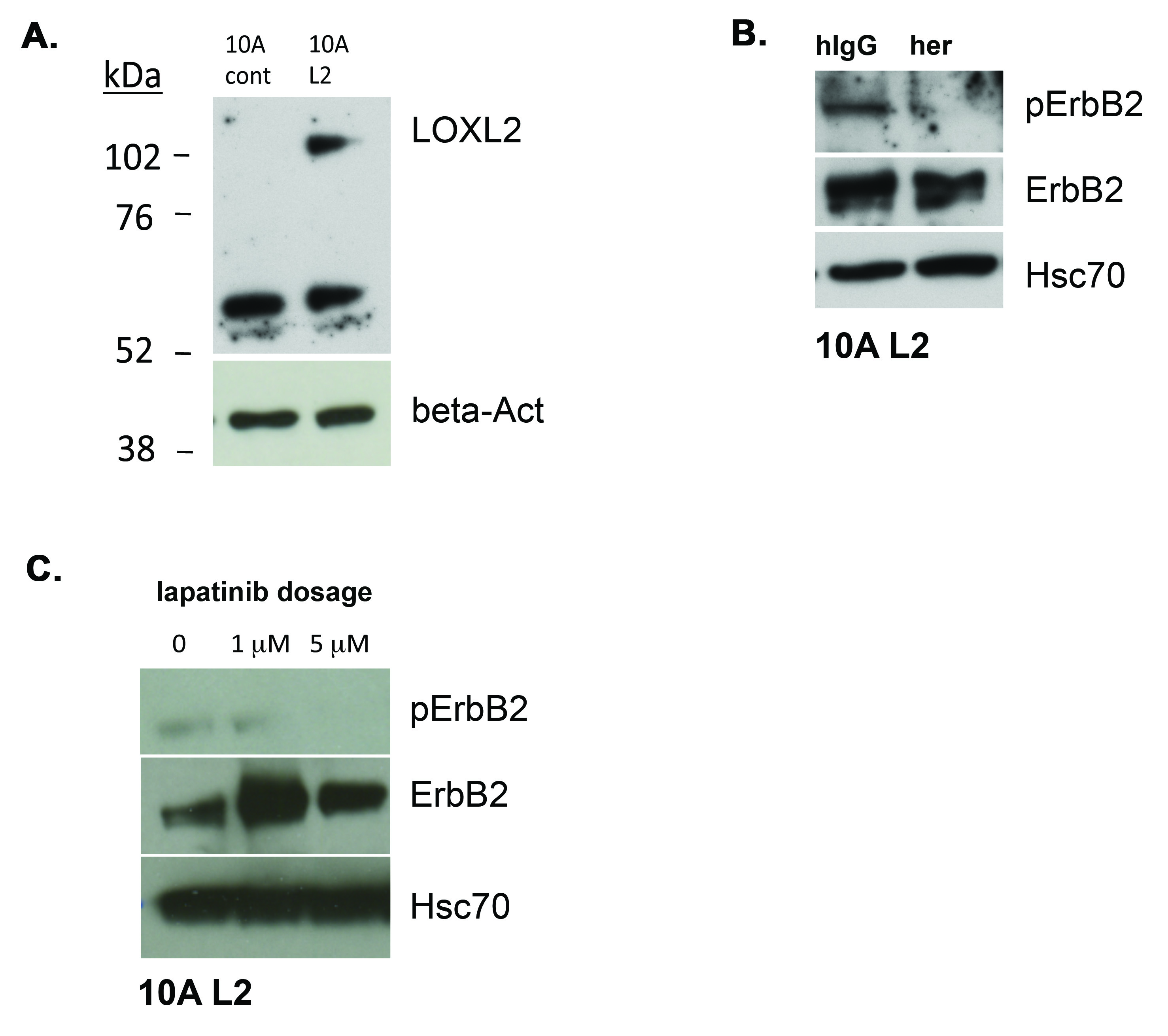

Supplement: Additional file 2 — Supplementary Figure S2. LOXL2 expression and signaling inhibition in MCF10A cells. (A) Western blot analysis of cell lysates from 10A cont and 10A L2 cells revealed that intracellular LOXL2 was also increased in the 10A L2 cells. (B) 10A L2 cells were subjected to overnight treatment with either 300 nM trastuzumab (Herceptin; L2+her) or the equivalent amount of human IgG (L2+IgG) followed by 3 hours of serum-starvation and 15 minutes of serum-blasting. Western blot analysis of the cell lysates revealed that at 300 nM, Herceptin inhibits the phosphorylation of ErbB2 in the 10A L2 cells. (C) 10A L2 cells were subjected to overnight treatment at the indicated dosage of lapatinib (0 μM (that is, DMSO only), 1 μM, and 5 μM) followed by 3 hours of serum-starvation and 15 minutes of serum-blasting. Western blot analysis indicated that lapatinib inhibits phosphorylation of ErbB2 at only 5 μM concentration. [file bcr3461-S2.JPEG]

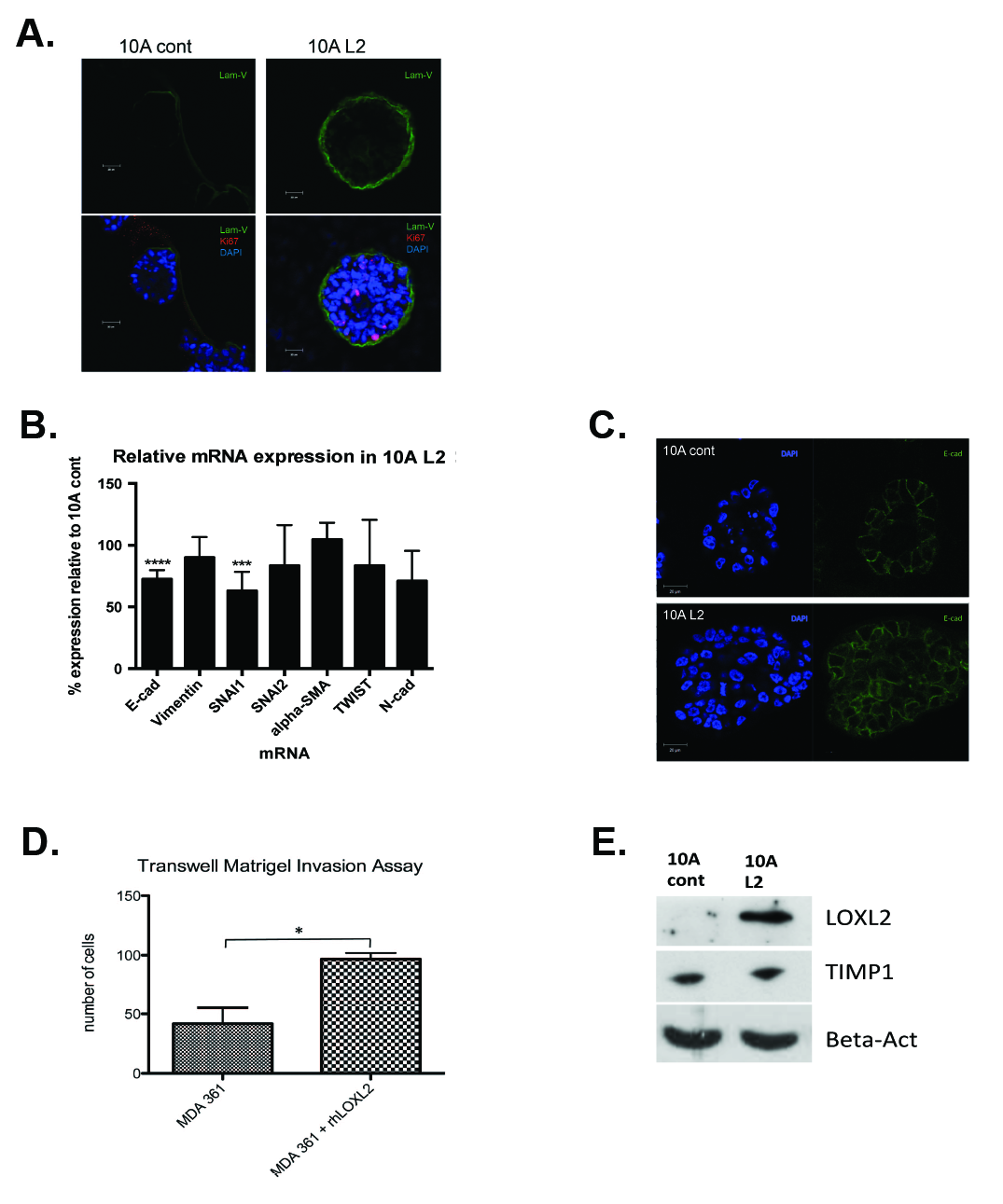

Supplement: Additional file 3 — Supplementary Figure S3. LOXL2 does not induce invasion or EMT in 10A acini, but increases invasiveness of MDA-MB-361 cells. TIMP-1 levels are not altered by LOXL2 expression. (A) Acini were cultured as described in Figure 2 and then fixed and stained with laminin V (Lam-V; Millipore) for the basement membrane and Ki-67. Representative photos from three independent experimental repeats are presented. Scale bar, 20 μm. Intact basement membrane, as evidenced by Lam-V staining, suggested that these cells do not form invasive structures when cultured in 3D. (B) Quantitative RT-PCR of Vimentin, E-cadherin, N-cadherin, SNAI1, SNAI2, Twist, and α-SMA in manipulated MCF10A cells showed that only E-cadherin and SNAI1 mRNA levels were downregulated in 10A L2 cells. Error bars represent SEM of three independent experiments. P = 0.00005 for E-cadherin and P = 0.00039 for SNAI1. (C) Acini were cultured as described in Figure 2 and then fixed and stained with E-cadherin (E-cad, Abcam). Representative photos from three independent experimental repeats are presented. Scale bar, 20 μm. 10A L2 acini did not have decreased E-cadherin protein levels. (D) The noninvasive ErbB2-amplified breast cancer MDA-MB-361 cells were subjected to Transwell invasion assays in the presence or absence of 50 nM rhLOXL2. Results indicated that recombinant LOXL2 increased the invasiveness of the noninvasive cells. P = 0.0176. (E) Western blot analysis of TIMP-1 levels in 10A cont and 10 L2 CM. Results indicate that TIMP-1 levels are unchanged between the two cell lines. [file bcr3461-S3.TIFF]

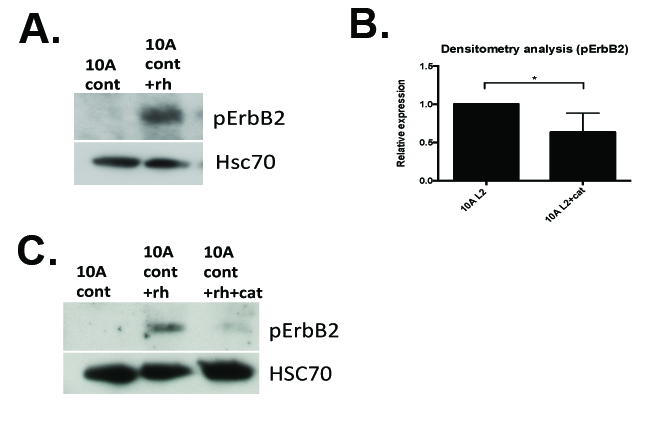

Supplement: Additional file 4 — Supplementary Figure S4. Recombinant human LOXL2 (rhLOXL2) rapidly activates ErbB2, and H2O2 depletion inhibits this activation. (A) The 10A cont cells were plated out, as described in Figure 3. After overnight incubation, cells were serum-starved for 3 hours and stimulated with 50 nM rhLOXL2 for 15 minutes. Western blot analysis revealed that ErbB2 was activated rapidly. Densitometry analysis was calculated on pErbB2 expression relative to Hsc70. (B) Densitometry analysis revealed that catalase treatment significantly decreased activation of ErbB2 in 10A L2 cells (P = 0.026) and was calculated on pErbB2 expression relative to ErbB2. (C) Catalase treatment (cont+rh+cat) abrogated rhLOXL2-mediated ErbB2 activation in 10A cont cells (cont+rh) (right blot, P = 0.0017 for cont versus cont+rhLOXL2; P = 0.043 for cont+rhLOXL2 versus cont+rhLOXL2+cat). Densitometry analysis was calculated on pErbB2 expression relative to Hsc70. [file bcr3461-S4.TIFF]

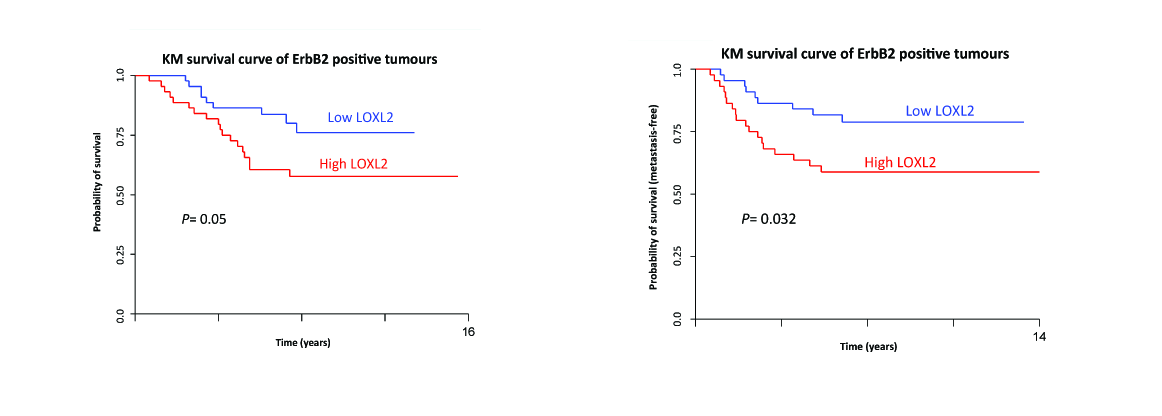

Supplement: Additional file 5 — Supplementary Figure S5. LOXL2 expression is correlated with metastasis in breast cancer patients with ErbB2+ tumors. Kaplan-Meier survival curves were constructed for patients with Her2/ErbB2-positive tumors by using MAS5 parameters, with global scaling set to 600 [19]. Overall survival (left panel; P = 0.05) and metastasis-free survival (right panel; P = 0.032) are shown here; n = 88 patients. [file bcr3461-S5.TIFF]
